# Identification of and Support for Children of Mentally Ill Parents: A 5 Year Follow-Up Study of Adult Mental Health Services

**DOI:** 10.3389/fpsyt.2018.00507

**Published:** 2018-10-16

**Authors:** Camilla Lauritzen, Charlotte Reedtz, Kamilla Rognmo, Miriam A. Nilsen, Anja Walstad

**Affiliations:** UiT The Arctic University of Norway, Tromsø, Norway

**Keywords:** implementation, changed clinical practice, children of mentally ill, mental health care for adults, children's perspective

## Abstract

**Background:** Children of parents with mental disorders are more likely to develop mental difficulties during their childhood and adulthood. Based on this knowledge, the Norwegian health legislation has been amended to better identify and protect children of parents with a mental illness. In this project, two interventions were implemented in a regional clinic for adult mental health services. These interventions were (i) Assessment Form and (ii) Child Talks. Both interventions aimed to support healthcare professionals in identifying and providing support for children of patients within adult mental health services. The process of changing relevant practice to become more family-focused was evaluated in 2010 and 2013, and the results showed some changes slowly materializing in the adult mental health services. The purpose of the current study was to investigate long-term effects of the interventions at 5 year follow-up (2015). The main aim was to investigate whether the workforce perceived that their clinical practice had changed as a result of the legislative change and the implemented interventions.

**Method:** This longitudinal study consists of a pre-test, post-test and follow-up test. The sample (*N* = 219 at pre-test, *N* = 185 by post-test and *N* = 108 on follow-up test) included healthcare staff from a participating hospital, responding to an online survey about their routines for identifying children of patients, their attitudes, as well as concerns and expectations related to having a child perspective in their clinical work. Employee experiences with family conversations were also investigated, as well as their knowledge about the consequences parents' mental disorders may have for children.

**Results:** Our findings showed a significant increase in participants identifying children of patients between pre- and post-measurement but a minor, non-significant increase at follow-up measurement. There was no significant increase of participants who reported that they had a lot of experience with family conversations. From post-test to follow-up, there was no increase in the workforce' reported positive attitudes, knowledge or expectations about the effects of the interventions.

**Conclusion:** There have been some changes in clinical practice, but it seems that the changes required by law are a very time consuming process. It is necessary to increase the pace of the implementation process.

## Introduction

Mental illness among parents is a risk factor that can have serious consequences for their children. In the course of the last two decades, findings from a number of studies have indicated that children of mentally ill parents have an increased risk of developing mental illness themselves (1–3). The risk for this outcome has been shown to vary between 41 and 77% (2, 4–7). Children of mentally ill parents are more vulnerable to depression, and compared with other children they are also more likely to develop reduced social skills, behavioral problems, hyperactivity, attention deficit disorders and lower self-confidence in their childhood and youth (8–10). In addition, children of mothers with depression have been shown to score lower on cognitive ability tests and perform worse in school (11). Furthermore, studies have shown that children of mentally ill parents have an increased risk of developing a variety of psychiatric disorders as adults, including depression, anxiety and substance abuse (9, 12).

A large number of children and youth live with parents who are suffering from mental illness, which is linked to the high rates of mental disorders in the population (13). Calculations carried out by the Norwegian Institute of Public Health in 2011 estimated that 410,000 children in Norway live with either one or two parents who have a mental illness, and 115,000 of these children had parents with serious mental illnesses (13). Another Norwegian study has shown that 13% of patients at 24 clinics and 33% of patients at psychiatric outpatient clinics were responsible for caring for children (14). These figures correspond to studies from other countries (15, 16).

It is assumed that the mechanisms that cause mental illness to be transmitted from parents to their children may be due to a number of different factors (17, 18). It is not the parents' diagnosis in and of itself that transmits this risk, but rather an interaction between factors such as disease severity and duration, genetics, coping style and social conditions (19). However, it is well documented that general parenting skills and the quality of the interaction between the parents and the child plays an important role in this transmission (20, 21). Among other things, it has been shown that this group may be less sensitive to their child's signals and needs (22, 23). Several studies can document a correlation between the quality of parenting skills and dysfunctional emotional regulation, insecure attachment and lower self-esteem (9, 22, 23).

In addition to interventions that are geared toward improving parenting skills, a growing literature has shown that the situation for children of mentally ill parents improves when they have the opportunity to talk about their parents' illness (24–26). In a large Norwegian multicentre study, researchers found that children of mentally ill parents have an unmet need for understanding their parent's condition, as well as its consequences (27), and it is not uncommon for the children to feel shame or guilt related to their parents' struggles (24). Interventions that aim to provide age-appropriate information to the children of mentally ill patients have been shown to help increase the children's comprehension of their parents' illness, improve communication within the family and enable the children to better master their situation when the parent is ill (24). Combined, these findings provide grounds for optimism about the results that can be expected if measures are implemented to provide children and parents with information about mental illness, and the opportunity to talk about the parents' mental illness.

In addition to the above benefits of preventing transmission of mental illness from one generation to the next, it has been argued that incorporating the child's perspective in psychiatric clinics also may provide additional health benefits for patients who are parents. An American study showed that mothers with mental illness identified their role as mothers as a primary factor in their treatment. They reported that not being able to raise and care for their children affected their mental health negatively, and prevented their progression toward improvement (28). Interventions that aimed to inform families about how depression affected the parents' functional level, and to provide strategies to detect and deal with stress, reduced symptoms of anxiety and depression in both parents with depression and their children (26, 29). A meta-analysis conducted by Pitschel-Walz et al. (30) reports that treatment that includes the children reduces relapses for patients with schizophrenia by up to 20%. Similarly, (31) found that family-focused interventions resulted in fewer relapses for those with bipolar disorder than in patients who only received medication and maintenance measures. Thus, it is evident that treatment approaches that include the patient's children and family can lead to positive outcomes and improve the situation for both the children and the patient.

Mental health care providers have a unique opportunity to identify children at risk through direct contact with the parents of the children in question. Incorporating a child's focus in mental health services for adults involves having an attitude that children and family are central parts of the patient's life that therefore must be taken into account in the treatment. This requires that health care providers incorporate the family in the treatment process, address the needs of the children and engage in the patient's parenting role (32). Clinics in mental health services for adults can be major contributors in the effort to provide the necessary help for children of the mentally ill. Central tasks include assessing the children, examining how they experience their situation, contributing information and psychoeducation, and taking steps to ensure further action if necessary (33). Norwegian health legislation has since 2010 obligated health personnel to identify whether the patient has children, and to help fulfill the children's need for information and follow-up if necessary.

### Challenges in clinical practice related to children of parents with a mental illness (COPMI)

Despite political guidance and the fact that the children of patients now have a statutory right to be identified and cared for, a number of studies have revealed challenges in the efforts to incorporate the child's perspective in mental health services for adults (27, 34, 35). These studies suggest that there is a gap between what health services *should* offer and what is *actually* offered in services for adults.

In studies conducted by Lauritzen et al. (34, 35), the findings indicated that some of these barriers were at the organizational level. The challenges were related to the high workload staff were under, a lack of ownership of the issue, as well as insufficient allocation of time and resources (35). Other barriers relate to the characteristics of the staff in the clinic. Research has shown that some of these challenges may have two sides. On the one hand, health care providers say that they believe it is important to support the children of patients, but that they at the same time do not consider this to be one of their tasks (35, 36). On the other hand, health care providers express concern about including the patient's children in the course of treatment (37). Furthermore, Lauritzen et al. (34, 35) results indicated that there are barriers related to health care providers' knowledge, lack of training and uncertainty. In this context, other studies have shown that experience and positive expectations related to working with children and the family have an important bearing on the implementation of a focus on children in clinical practice (32).

### The norwegian copmi project

The COPMI project is a longitudinal research project in which the goal was to support the implementation of new routines arising from legislative amendments, as well as to evaluate the process of change. The project started in 2010, and involves a long-term strategy for changing clinical practice. The current study is a five-year follow-up study.

The aims of the current study were to investigate:

- Has the proportion of health care providers identifying patient's children increased, 5 years after the legal changes?
- Has the proportion of health care providers reporting that they support the patient's role in parenting and safeguarding the patient's minor children changed over time?
- Has the implementation of the new routines led to higher levels of employee's expectations of positive outcomes, attitudes and knowledge about children as next of kin?
- Has the implementation of the new routines led to lower levels of health care providers' concerns related to assessments of whether the patient/therapist relationship will be disturbed by the focus on the patient's children?
- Are there differences in expectations, knowledge, concerns and attitudes between employees who have little experience with family conversations and those who have a great deal of experience?

## Methods

### Participants

In the pre-study, 219 employees responded (50% response rate), whereas in the post-study, 185 employees responded to the email, corresponding to a response rate of 40.5%. In this follow-up study, 108 employees participated (24.5% response rate). Demographic information is presented in Table 1.

**Table 1:** Study sample: descriptive statistics of the study sample and result of analysis of variance of sample characteristics according to measurement point.

	**Pre-test (*N*^total^ = 219)**	**Post-test (*N*^total^ = 185)**	**Follow-up (*N*^total^ = 108)**	
	***N* (%)**	***N* (%)**	***N* (%)**	***p***
Sex				0.171
Women	166 (76.1)	123 (71.1)	76 (74.5)	
Men	52 (23.9)	50 (28.9)	26 (25.5)	
Age				0.525
< 30	25 (11.5)	11 (6.0)	6 (5.6)	
31–40	55 (25.2)	38 (20.7)	22 (20.6)	
41–50	57 (26.1)	65 (35.3)	36 (33.6)	
51–60	60 (27.5)	51 (27.7)	31 (29.0)	
>60	21 (9.6)	19 (10.3)	12 (11.2)	

The participants were health care providers employed in the adult psychiatric clinic at the University Hospital of North Norway. The clinic consisted of a total of 16 outpatient clinics, and included a geographical area consisting of 31 municipalities. Participants were recruited through a written invitation sent to their email address, in which they were asked to answer online questionnaires (Quest-Back). It was the clinic leadership that made lists of the employees' email addresses available to the research group. The participants were informed that participation was voluntary, and that they would remain anonymous. As a result of the participants' anonymity, there were independent selections in the pre, post, and follow-up studies. The study was approved by the data protection supervisor at the University Hospital of North Norway, and was carried out in accordance with the Declaration of Helsinki on ethical guidelines for medical research involving people (38).

### Procedure

The clinic initiated new procedures to identify and offer the necessary follow-up for children of psychiatric patients and this work included the implementation of two interventions: *Assessment Form* and *Child Talks*. The *Assessment Form* is an intervention for treatment providers to increase the identification of patients' minor children. The Assessment Form consists of a general section where information that the providers were required to collect as a result of legislative changes is requested (for example, the child's name, age, siblings, living situation, etc.) and a section that maps the parents' concerns and skills related to parenting. The *Child Talks* is an intervention designed to provide support for patients with mental illnesses who are parents and for their children. The purpose is for the talks to promote health and prevention by having the treatment provider speak with the family about the children's situation and their needs, as well as to provide support to the family and assist them in seeking help from other health services if necessary (39).

### Measures

#### Routine for identification

The employees' routine for identifying children was measured through the test item: “Have you completed the Assessment conversation?” Participants were asked to answer *Yes* or *No*. Due to miscoding, some responses were recoded into missing values (Pre-test N recoded = 3, Post-test *N* recoded = 2).

#### Family conversations

Experiences with the family conversations were measured through the test item “To what extent do you have experience with family conversations?” Participants were asked to rank their response on a five-point Likert scale, from “To a very small extent” (1) to “To a great extent” (5). Experience with family conversations was also used as a predictor variable in one set of analyses. When used as a predictor, the variable was categorized into “low experience,” “neither low nor high experience,” and “high experience” with family conversations.

#### Expectations about positive outcomes

Participants' expectations about the effect the new routines may have for the children of patients was measured using a scale consisting of four test items. The scale was calculated as the average score of the four test items. Cronbach's alpha for this scale was 0.91 (calculated with re-coded variables without 0 responses). Test items included questions about expected outcomes for patients and their children, and one example of the questions is “I think conversations about and with children can help improve the life situation of children of parents with a mental illness.” Participants were asked to rank their response on a five-point Likert scale, from “To a very small extent” (1) to “To a great extent” (5).

#### Knowledge

Participants' knowledge of the children of mentally ill parents was measured using questions retrieved from the *Family focused mental health practice questionnaire* (40). The questionnaire was adapted to Norwegian conditions/contexts with the permission of the authors. The nine test items included both questions that dealt with knowledge about the children and questions related to knowledge about the new legislation. One example question is “To what extent do you have knowledge about mental illnesses and the consequences such disorders may have for the parenting role.” Participants were asked to rank their response on a five-point Likert scale, from “To a very small extent” (1) to “To a great extent” (5).

A factor analysis was conducted to reduce the number of test items. Data was considered to be suitable for factor analysis, with a KMO value of 0.86 and Bartlett's test of sphericity was significant. A Principal Component Analysis (PCA) with Varimax rotation indicated that all test items, with the exception of one, were high loading on one factor. This test item was “To what extent have you received training in the new legislation?” This test item was thus excluded, and a scale was calculated based on the average score of the eight remaining test items. The scale was named Knowledge, and Cronbachs alpha for this scale was 0.86.

#### Attitudes

The ten test items included questions related to the willingness to change within the organization, attitudes to the new practice and attitudes to the importance of having a focus on the children of mentally ill parents. In addition, the test items related to attitudes about whether or not a child perspective in the practice interferes in the therapeutic alliance with the patient. Test items included questions such as “We should offer the children of patients information and support as next of kin,” and “The relationship between the treatment provider and patient may be negatively impacted if the patient's parenting role is brought up.” Participants were asked to rank their response on a five-point Likert scale, from “To a very small extent” (1) to “To a great extent” (5).

A factor analysis was conducted to reduce the number of test items (re-coded data without 0 responses). The estimated KMO value was 0.88 and Bartlett's test of sphericity was significant, which indicated that the data was well suited for factor analysis. A Principal Component Analysis with Varimax rotation revealed two components. Positive attitudes to a focus on patients' children was high loading on component 1, while negative attitudes associated with concern about disruption in the treatment and interference in the parenting role was high loading on component 2. The interpretation of the two components is consistent with previous research for the scale associated with Attitudes (34). Two scales were calculated based on the average scores for the test items in the two components. The two scales were named Positive Attitudes and Concerns, and the reliability analysis calculated Cronbachs alpha to be 0.94 for Positive Attitudes and 0.79 for Concerns.

#### Demography

Age and sex were also reported in the questionnaire.

### Procedure

The treatment provider was to conduct a family assessment conversation and fill out a form together with the patient, and should then enter it into the electronic patient record. The family assessment consists of questions such as “Do you have children?,” “How many children do you have?,” and “Do you have the daily care of the child?.” At the end of the conversation, the treatment provider should inform the patient about the Child Talks, and offer this to the patient (41). If he or she agreed to participate in Child Talks, the talk would normally be held in the course of the next 4 weeks.

Data collection was carried out electronically using email through Quest Back on three different measuring points in pre (2010), post (2013), and follow-up (2015) studies. Participants received an email in which they were asked to answer an online questionnaire. If they did not respond, Quest Back generated reminders about the study after 2 weeks. This was done for a maximum of three intervals. This article reports the results from the follow-up study, where we wanted to examine whether the implementation of the two interventions (Family assessment and Child Talks) had led to a change in clinical practice 5 years after health legislation was altered to make assessment and support mandatory for families affected by parental mental illness.

### Data analyses

The data was exported from Quest Back to SPSS. All statistical analyses were performed in IBM SPSS (version 24). Descriptive analyses were used to examine the demographic characteristics of the group. Hierarchical logistic regression analysis was used to test the probability of having identified children of patients, in a two step manner. First the crude odds ratios were estimated, and next the estimates were adjusted for sex and age. Multivariate analyses of variance were used to test the degree to which familiarity with family conversations, knowledge, expectations, positive attitudes, and concern varied according to measurement point. The estimates were adjusted by sex and age. Similar multivariate analyses of variance were conducted to test the degree to which knowledge, expectations, positive attitudes and concern varied according to experience with family conversations. Once again, sex and age were adjusted for.

## Results

### Assessment and identification

There was a significant increase in the proportion of participants who identified the patients' children between the three points at which measurements were taken. In the pretest measurement, 44% of participants reported that they had used the Assessment Form, while at the posttest stage, 66% reported that they had used it. At the follow-up test, the proportion had again increased to 72% of the participants. Results are displayed in Table 1.

The results indicated that the different measurement points did have an impact on the probability that the employees used the Assessment Form, and thus identified the patients' children. Both the adjusted and unadjusted estimates showed a significantly greater probability of having used the Assessment Form at the post-test (adjusted OR = 2.29) and at follow-up (adjusted OR = 3.37) compared to the pre-test. The confidence intervals between the posttest and follow-up test were overlapping, indicating that there was no significant difference between these two measurement points. See Table 2 for an overview.

**Table 2:** Logistic regression analysis of the probability of identification of children of patients according to measurement time (pre-test, post-test and follow-up).

		**Model 1^a^**			**Model 1^b^**		
	***N*^yes^ (%)**	***OR***	***CI***	***p***	***OR***	***CI***	***p***
Pre-test	95 (44)	Ref.^c^			Ref.^c^		
Post-test	120 (65.6)	2.32	1.53–3.51	< 0.001	2.29	1.51–3.74	< 0.001
Follow-up	73 (71.6)	3.37	2.00–5.70	< 0.001	3.37	1.99–5.71	< 0.001

a *Unadjusted estimates*.

b *Estimates adjusted by sex and age*.

c *Reference group*.

### Experience with family conversations

In order to examine whether more health care providers had experience with family conversations at the post-test and follow-up test compared to the pre-test, a multivariate analysis of variance was run. The estimates were adjusted for sex and age. The results revealed no significant difference [*F*_(2, 467)_ = 1.085, *p* = 0.339]. The average value and standard deviation for the scores are presented in Table 3.

**Table 3:** Multivariate analyses of variance of differences in having performed Family conversations, Knowledge, Expectations, Positive attitudes, and Concerns according to measurement time (pre-test, post-test, and follow-up), adjusted by sex and age.

	**Pre-test (*N* = 203–215)^a^**	**Post-test (*****N*** = **176–182)^a^**			**Follow-up (*****N*** = **99–105)^a^**			**Total**
	***B***	***B (CI)***	***SE***	***p***	***B (CI)***	***SE***	***p***	***p***
Family conversations	Ref^b^	0.15 (−0.03 to 0.36)	0.10	0.143	0.56 (−0.19 to 0.30)	0.12	654	0.339
Knowledge	Ref^b^	0.18 (0.07 to 0.30)	0.06	0.001	0.16 (0.02 to 0.29)	0.07	0.022	0.003
Concerns	Ref^b^	0.10 (−0.05 to 0.25)	0.08	0.188	0.17 (−0.01 to 0.35)	0.09	0.063	0.143
Positive attitudes	Ref^b^	−0.25 (−0.38 to 0.11)	0.07	< 0.001	−16 (−0.32 to 0.01)	0.08	0.044	0.001
Expectations	Ref^b^	−0.14 (−0.26 to 0.01)	0.06	0.031	−0.12 (−0.27 to 0.03)	0.07	0.104	0.067

a *The variability in N is caused by missing values for some of the variables*.

b *Reference group*.

### Knowledge, positive attitudes, concerns and expectations

Multivariate analyses of variance were conducted in order to estimate the degree to which the participants in the pre, post and follow-up groups scored differently on the outcome variables Knowledge, Positive Attitudes, Concerns, and Expectations. The estimates were adjusted for sex and age. The results of the multivariate analyses are presented in Table 3. There was a significant difference in the average score for Positive Attitudes between the participants in the three groups (*p* = 0.002). The mean score on positive attitudes were lower both at the post-test (*B* = −0.25, *p* < 0.001) and at follow-up (*B* = −0.016, *p* = 0.044) than at the prtest.

There was also a significant difference in Knowledge between participants at the three measurement points (*p* = 0.003). Knowledge was significantly higher both at the post-test (*B* = 0.18, *p* = 0.001) and at follow-up (*B* = 0.16, *p* = 0.022) compared to at pre-test. Overlapping confidence intervals indicated that there was no significant difference between the post-group and the follow-up group. There were no significant differences in reported Expectations or Concern between the participants in the pre, post and follow-up groups.

### High and low experience with the family conversations

Furthermore, we wanted to investigate whether there were differences between health care providers who had a high and a low degree of experience with family conversations, in regards to Knowledge, Positive Attitudes, Concerns and Expectations of positive outcomes. The multivariate anova, with adjustments for sex and age, showed a significant difference only in Knowledge [*F*_(2, 460)_ = 154.253, *p* < 0.001]. The group with low experience with family conversations had on average −0.93 (*p* < 0.001) lower scores on Knowledge, whereas the group with neither high nor low experience had on average −0.54 (*p* < 0.001) lower scores on Knowledge, compared to the reference group with high experience with Family conversations. Level of experience with family conversations was unrelated to Positive attitude, Concerns and Expectations.

## Discussion

The objective of this follow-up study was to examine whether there have been permanent changes in clinical practice related to assessing and supporting children of patients in units for mental health services for adults at UNN. The results indicated that some changes have been integrated since the project's start-up, however health care providers had not changed their practices in such a way that all children of patients are identified and offered support. The results further indicated that after the project withdrew from the participating hospital in 2013, there has not been sufficient systematic work around assessing and safeguarding the need for information that children of mentally ill patients have.

Our findings indicated that the biggest changes happened in the first few years after the legal changes came into effect and at the time the implementation process started up at UNN. There was a significant increase in the number of employees who reported that they identified the children of patients after the Assessment Form was implemented in 2010: the proportion rose from 44% in the pre-study to 66% in the post-study in 2013. Subsequently, there was a smaller, non-significant increase until 2015, when 72% of employees reported that they implemented the required form and identified the patients' children. The results showed that 28% of the participants still do not assess if patients have children. The registration of patients' children is a key part of the 2010 legislation, and after the project withdrew in 2013 there has not been any statistically significant increase in the number of participants who identify if patients are parents.

A successful implementation of the Assessment Form may in this context be considered a quality indicator (27), as it accords with the health authority's guidelines and provides a thorough review of the circumstances such as the child's name and age, as well as clarifications about the patient's care responsibilities. Unfortunately, our results indicated that the implementation had not been successful, as a high proportion of the participants report they are not using the Assessment Form. One possible explanation could be that they register patients' children in other documents in the patient journal (42). If so, this is a less systematic documentation of patients' children than the Assessment Form, and the degree of information registered in the record may vary between treatment providers.

An even greater challenge has related to implementing the Child Talks in the clinical practices of health care providers at the hospital. The results indicated that there has not been any significant increase in the employees' experience with family conversations since the implementation process started in 2010. At that time, 20% of the employees reported that they had high experience with family conversations, while in 2013, 23% of the participants said the same. In 2015, 26% reported having a high experience of these conversations. It is possible that “family conversations” meant something different to the employees at the last two measurement points than it had at the pre-measurements. In 2010, the implementation of the Child Talks had not yet started up, and it is therefore possible that the reported experience of family conversations had a more general meaning and was not necessarily linked to this specific intervention. One would still expect that if more employees had begun to implement the Child Talks after the implementation started in 2010, participants would also have reported higher levels of experience with family conversations in 2015, which does not seem to be the case. We therefore do not think that different perceptions of what the family conversations are is a plausible explanation for the fact that 75% of the employees still report low levels of experience related to talking with the patients' families, even five years after the new law required health care providers to support and safeguard the information needs of children of mentally ill patients.

However, our findings still support the claim that the employees value a focus on children in the clinical practice. Participants generally had a high degree of positive attitudes to the introduction of the Child Talks in the clinical practice, which is reflected in the high average scores at all three measuring points. However, participants were the most positive to the introduction of a child's perspective in 2010, the same year that the new law came into force. The participants reported a significantly lower degree of Positive Attitudes in 2013, while there was no significant change between the measurements in 2013 and 2015. The participants reported that they largely agreed that health care providers should identify the children of patients, offering them information and support, and initiate a dialogue about the parenting role with patients who have children. The results indicated that there is no significant change between the three measurement points in the employees' concerns related to having a focus on patients' children in the treatment. The participants were generally not very concerned that talking with patients about their children and parenting role may damage the patient/therapist relationship, which is reflected in the low scores for concern. The employees' positive attitude as well as the low level of concern, may indicate that there is a certain degree of willingness to change among the employees (43). Despite this, more than 25% of the participants still report that they do not seek to clarify whether the patients have children, and more than 75% have little experience with talking to the patient's children at the follow-up point in 2015. The results thus indicate that a significant proportion of the staff has not integrated the new procedures in their clinical practices, despite the fact that they show a high degree of positive attitudes to including a focus on the patient's children. This is in line with the findings from the pre and post study in the project (34, 35), as well as past research (36, 44), which indicates that the positive attitude of the staff is not sufficient for them to incorporate a focus on patients' children in the treatment.

We also wanted to investigate whether there has been any change in the employees' level of knowledge since the implementation of the new interventions started in 2010. The results show a significant increase in participants' self-reported knowledge from the implementation process started in 2010 and to it reached its highest level of activity in 2013. There was no change in participants' knowledge from 2013 to 2015, which indicates that the employees' level of knowledge has remained stable after the project withdrew from the hospital. However, it is possible that the reported increase in knowledge between the measuring points in the pre and post study is not very significant in the clinical practice. Although there was a statistically significant change between the measuring points in 2010 and 2013, the calculation of the effect size showed that this was virtually negligible.

The results from this survey indicate that there also was no difference between these two groups related to concerns or positive attitudes. Both those with high and low degrees of experience with the family conversation had a very high degree of positive attitudes to focusing on the patient's children during the treatment. However, our results did indicate a difference in the reported level of knowledge between the two groups. Employees who had more knowledge about children of mentally ill parents also reported more experience with family conversations than those with lower levels of knowledge. This can be seen in the context of discoveries made in the measurements carried out in 2010 by Lauritzen et al. (34). This finding showed that employees who reported that they identified the children of patients also had a higher level of knowledge about children as next of kin and of the legislation than those who did not identify the children at this measurement point. These findings are also consistent with results from studies that have shown that the ability to support patients in the parenting role increased through the health care provider's personal experience and their professional experience and education in this area (45, 46). On the other hand, studies have revealed that a lack of knowledge and skills can be obstacles to the implementation of the child's perspective in clinical practice (44).

There are reasons to assume that there is potential to strengthen the training of health care providers, so as to increase their level of knowledge and skills related to working with children as next of kin. The results from a Norwegian multicentre study showed that 40% of the employees in mental health services for adults had not participated in training related to practizing the new legal provisions (27). In this context, Maybery and Reupert (44) propose that the training should have a two-part focus. One part should be directed toward the patient's parenting role, and should provide knowledge of parental guidance and increase the ability to discuss the consequences that mental illness can have for the child in an empathetic way. The second part should deal with the direct work with children, where the ability to talk with children and provide age-appropriate information should be key (44). Another suggestion might be to include knowledge about work with children and families in the education programmes for the various health professions (27, 35), as this can give future health care providers an expanded and solid knowledge that increases their sensitivity to the children of patients once they enter the workforce.

### Child perspective team

It is not uncommon for innovations to have to be changed in order to adapt to the practices they are to be implemented in Meyers et al. (47), and it is possible that it is necessary to rethink the implementation of the family conversations in the participating hospital. A strengthened partnership between the adult and child services can help promote the safeguarding of patients' children and families (27). In addition, the interdisciplinary cooperation between different health services is important in the efforts to offer support to children as next of kin (27, 48). By involving key personnel, who are often referred to as “champions” in the literature, conditions can be created that promote the implementation process (47). The champions have a high level of knowledge and motivation, as well as the ability to engage the rest of the staff (47). Champions can uncover barriers that delay the implementation process in the clinic, and help develop solutions to address these obstacles (48). It has previously been proposed that an employee with responsibility for children can fulfill such a role in the clinic (48), but this requires that more time and resources be set aside for this work (33). Furthermore, the employee with responsibility for children must be anchored at a sufficiently high level in the organization, and must be given influence in the clinical practice (27).

With regard to the identification and assessment of the patients' children, this will still be the responsibility of the patient's treatment providers, as is done in the existing routines. Possible measures to increase the identification and registration of patients' children may be to provide adequate training in keeping patient records for health personnel (27), and/or to introduce the registration of patients' children as a quality indicator for the hospital's service (42).

## Conclusion

The children of mentally ill parents is a group with a known risk of developing mental illness, and since 2010 they have had a statutory right to be identified and cared for by health care providers in Norway. The interventions “Assessment Form” and “Child Talks” comply with the statutory requirements, and can prevent and reduce the risk that children of patients themselves develop mental illnesses. Our results show that the hospital has not managed to implement changes in its practices to ensure that all minor children of mentally ill patients are assessed and cared for in a satisfactory manner. On this basis, we have proposed specific measures to establish dedicated teams at psychiatric centers within the hospital, which will be responsible for conducting conversations with the patient's children. Furthermore, we believe that the key personnel in such teams can help to overcome important obstacles at several levels in the organization, and speed up the implementation of the new procedures. However, this will be difficult to implement without adequate allocation of time, resources, and personnel.

## Limitations of the study

The most central limitation in this study is the relatively low response rate in the study, particularly at the follow-up point (50% for the pretest, 40% for the posttest and 24% for the follow-up measurement). This can affect the results if the decision to participate in the survey relate to or are affected by the respondent's attitudes. It is also possible that those who choose to answer the survey already have more positive attitudes attached to having a family perspective in the treatment they practice. One consequence of this may be that this article represents more positive attitudes in health care providers about the topic than what actually is the case. However, a response rate of 24% is quite common for online surveys. The cohorts of respondents did not differ significantly in terms of age and gender at the different times of measurement. Confront Table 1 for information about the studied population in terms of age and gender.

Another limitation of this study is the way some of the questions are worded in the questionnaire. Participants are asked whether they to a high or low degree have “experience with family conversations.” In our study, we are interested in examining whether the employees have begun to make use of the “Child Talks” intervention. It is possible that the participant interprets experience of family conversations in a larger context and is not responding specifically to whether they have experience with the “Child Talks.” Subsequent studies should therefore examine whether health care providers have started to make use of the “Child Talks.”

This study is based solely on measurements from self-reporting about attitudes, knowledge, expectations and current work practices. Future studies should also include objective measurements such as patient record data to investigate how many patients' records report on assessments of family conversations.

The samples at pre, post and follow-up tests are not independent, due to the anonymity of the respondents. The respondents may therefore have been influenced by being nested within the same department, which may have biased the results. In future research personnel should be evaluated individually to track changes in levels of competence and clinical practice.

In terms of the existing evidence for these interventions, there are few studies available. The interventions are being tested in Norway and Portugal, and we expect results about their effects to be published eventually. However, there is generally a lack of intervention studies that document the positive effects of incorporating a focus on the patient's children in clinical practice and treatment within the mental health services. Hence, future studies should be conducted to study effects of interventions that aim to promote well-being among children of parents with a mental illness. This may motivate hospital management and health care providers to make use of such interventions to a greater extent.

## Ethics statement

The Data Protection Officer at the University hospital of Northern Norway approved the project.

## Author contributions

All authors agree to be accountable for the content of the work. CL and CR designed the project. CL collected the data. Analyses were conducted by KR, MN, AW, and CL. CL drafted the article. All authors participated in the writing of the manuscript.

### Conflict of interest statement

The authors declare that the research was conducted in the absence of any commercial or financial relationships that could be construed as a potential conflict of interest.
